# Draft genome sequences of *Streptomyces virginiae* strain CMAA1738, *Paenibacillus ottowii* strain CMAA1739 and *Pseudomonas inefficax* strain CMAA1741, isolated from rhizosphere of wheat landraces

**DOI:** 10.1128/mra.00036-24

**Published:** 2024-06-11

**Authors:** Caroline Sayuri Nishisaka, João Paulo Ventura, Hélio Danilo Quevedo, Fernanda de Almeida Godoy, Maike Rossmann, Rodrigo Mendes

**Affiliations:** 1 Embrapa Environment, Brazilian Agricultural Research Corporation, Jaguariúna, Brazil; 2 Luiz de Queiroz College of Agriculture, University of São Paulo, Piracicaba, Brazil; University of Strathclyde, Glasgow, United Kingdom

**Keywords:** rhizosphere, microbiome, *Pseudomonas*, *Streptomyces*, *Paenibacillus*, genome

## Abstract

In this study, we have identified and characterized three genomes from bacteria isolated from the rhizosphere of *Triticum aestivum. Streptomyces virginiae* CMAA1738 and *Paenibacillus ottowii* CMAA1739 were obtained from the wheat landrace Iran 1-29-11334, and *Pseudomonas inefficax* CMAA1741 was isolated from the wheat landrace Karakilcik.

## ANNOUNCEMENT

Previous microbiome analysis comparing wheat landraces with modern cultivars revealed that landraces exhibit an enhanced ability to recruit specific microbes in the rhizosphere (1). Therefore, we selected two wheat landraces for bacterial isolations: *Streptomyces virginiae* CMAA1738 and *Paenibacillus ottowii* CMAA1739 were obtained from the Iranian wheat landrace Iran 1-29-11334, and *Pseudomonas inefficax* CMAA1741 was isolated from the wheat landrace Karakilcik, originally from Turkey.

For rhizosphere sampling, plants were grown in 250 mL-pots using soil collected from a wheat field in Brazil (22°55′45.36′S; 50°07′22.33′W) in 2017. Pots were kept in a growth chamber with16-h light/8 h dark cycle maintaining temperatures ranging from 20.7°C to 26.1°C. Plants were removed from the pots and gently shaken to remove loose soil; the soil adhered to the roots was sampled for bacterial isolation. One gram of rhizosphere soil was diluted in 9 mL of saline solution (8.5 g L^−1^ NaCl) and subjected to serial dilutions. Then, 100 µL of the 10^−3^, 10^−4^, and 10^−5^ dilutions were plated on trypticase soy agar medium. After incubation at 28°C for 96 h, colonies underwent purification through three to five consecutive streaks.

For DNA extraction, *S. virginiae* was cultivated in yeast malt extract agar medium at 28°C for 48 h. *P. ottowii* and *P. inefficax* were cultivated in glucose yeast medium at 28°C for 24 h. DNeasy Ultraclean Microbial kit (Qiagen, Germantown, USA) was used for DNA isolation. After quantity (Nanodrop ND-2000 Spectrophotometer - Thermo Fisher Scientific, Wilmington, DE, USA) and purity (QUBIT 2.0 - Thermo Fisher Scientific, Wilmington, DE, USA) checks, DNA samples were sent to Novogene (Novogene Corporation INC, El Monte, California, USA) for genome sequencing.

The whole-genome sequencing was performed using Illumina NovaSeq 6000 platform. Genomic DNA was used for paired-end libraries (2 × 150 bp) construction using NEBNext Ultra DNA Library Prep Kit for Illumina (NEB, USA) following manufacturer’s recommendations. Then, PCR products were purified with AMPure XP system (Beckman Coulter Life Sciences, Indianapolis, USA) and analyzed for size distribution by Agilent 2100 Bioanalyzer (Agilent Technologies, CA, USA). FastQC v0.12.1 (2) was used for sequences’ quality check, and Trimmomatic v.036 (3) was used for filtering, followed by genome assembly using SPAdes v3.15.3 (4). Quast v5.0.2 was used to measure the assembled genomes’ quality metrics (5, 6). The CheckM v1.0.18 (6) was used to assess sequence completeness and contamination. Bowtie2 v1.7.0 tool was applied to get alignment statistics of genomes (7). The taxonomy of each bacterium was determined using the Average Nucleotide Identity (ANI) method, with the assistance of the Genome Taxonomy Database toolkit (GTDB v1.7.0) (8–10). RAST v1.073 pipeline (11, 12) and Prokaryotic Genome Annotation Pipeline (PGAP) v6.6 (13) were used for genome annotation. Functional prediction for secondary metabolite production was assessed using antiSMASH v7.0.1 (14). Genome assembly statistics and additional information are summarized in Table 1.

**TABLE 1:** Summary of bacterial genomes assembly statistics and other information

Genomic profile	*S. virginiae*	*P. ottowii*	*P. inefficax*
Strain ID	CMAA1738	CMAA1739	CMAA1741
Fast ANI placement (%)*^a^*	98.19	96.60	97.83
Fast ANI reference (NCBI)^*b*^	GCF_014648795.1	GCF_006874425.1	GCF_900277125.1
Library size (millions of paired-reads)	51.77	47.59	51.57
Number of contigs^*c*^	113	72	294
Total sequence length (pb)^*c*^	8,104,969	5,672,255	5,652,986
Total ungapped length (pb)^*c*^	8,103,135	5,671,970	5,652,597
N50 (kb)^*c*^	167.1	312.3	36.5
GC (%)	72.50	45.00	63.00
Genes^3^	7,316	5,319	5,089
Protein-coding^*c*^	7,138	5,194	4,993
Non-coding (RNA)^*c*^	74	44	63
Genome completeness/ contamination (%)^*c*^	100.00/0.57	99.72/0.56	100.00/0.54
Coverage (x)^*c*^	1.02	1.20	1.33
SRA identifiers	SRR18100379	SRR18099329	SRR17914816
Genome assembly (NCBI)	GCF_035853655.1	GCF_035853635.1	GCF_035853745.1
RAST annotated genome and antiSMASH data	https://zenodo.org/records/10854839	https://zenodo.org/records/10854801	https://zenodo.org/records/10854821

^*a*^ Considered bacterial species demarcation criterion (>95%–96%) (15).

^*b*^ Closest related strain identified to date.

^*c*^ Calculated using NCBI PGAP.

## Data Availability

The genomes of *Streptomyces virginiae* CMAA1738, *Paenibacillus ottowii* CMAA1739, and *Pseudomonas inefficax* CMAA1741 are accessible at NCBI GenBank under BioProject numbers PRJNA802715, PRJNA802713, and PRJNA802578, respectively, with BioSample accession numbers SAMN25582084, SAMN25581302, and SAMN25556618. The SRA identifiers, genome assembly details and antiSMASH data are described in Table 1. The links for genome annotation using Rapid Annotations using Subsystems Technology toolkit (RAST) are available in Table 1. The authorization for field soil sampling is registered with the Brazilian National System for the Management of Genetic Heritage and Associated Traditional Knowledge (SISGen) under number A5EB05F.
